# Contaminating DNA in human saliva alters the detection of variants from whole genome sequencing

**DOI:** 10.1038/s41598-020-76022-4

**Published:** 2020-11-06

**Authors:** C. A. Samson, W. Whitford, R. G. Snell, J. C. Jacobsen, K. Lehnert

**Affiliations:** School of Biological Sciences, Centre for Brain Research, The University of Auckland, Auckland, New Zealand

**Keywords:** Corneal diseases, Vision disorders, Preventive medicine

## Abstract

Cells obtained from human saliva are commonly used as an alternative DNA source when blood is difficult or less convenient to collect. Although DNA extracted from saliva is considered to be of comparable quality to that derived from blood, recent studies have shown that non-human contaminating DNA derived from saliva can confound whole genome sequencing results. The most concerning complication is that non-human reads align to the human reference genome using standard methodology, which can critically affect the resulting variant genotypes identified in a genome. We identified clusters of anomalous variants in saliva DNA derived reads which aligned in an atypical manner. These reads had only short regions of identity to the human reference sequence, flanked by soft clipped sequence. Sequence comparisons of atypically aligning reads from eight human saliva-derived samples to RefSeq genomes revealed the majority to be of bacterial origin (63.46%). To partition the non-human reads during the alignment step, a decoy of the most prevalent bacterial genome sequences was designed and utilised. This reduced the number of atypically aligning reads when trialled on the eight saliva-derived samples by 44% and most importantly prevented the associated anomalous genotype calls. Saliva derived DNA is often contaminated by DNA from other species. This can lead to non-human reads aligning to the human reference genome using current alignment best-practices, impacting variant identification. This problem can be diminished by using a bacterial decoy in the alignment process.

## Introduction

In recent years, high-throughput genome sequencing has become an essential research and clinical tool. The applications of genome sequencing are broad, ranging from revealing personalised treatment options to the diagnosis of genetic disorders^1–5^. Blood is often the preferred DNA source for genome sequencing. However blood collection can be a challenge particularly for neonates or individuals with developmental or behavioural conditions, as blood collection is more invasive than saliva collection and potentially distressing due to the use of needles. Alternatively, genomic DNA can be extracted from saliva, which is simpler to obtain, can be self-collected, and is stable in the collection device at room temperature for extended periods without degradation (> 5 years)^6–8^. This method of DNA collection has become increasingly popular over the last ten years due to ease of collection and improvements in the quality and quantity of DNA that can be collected from saliva^9,10^. Collection of saliva DNA is commonly used for population studies and to obtain samples from remote areas^11–15^. The accuracy of genotyping in saliva-derived DNA has been reported as comparable to DNA derived from blood^16–18^.

There are potential complications associated with the use of saliva as a DNA source. DNA extracted from saliva may contain non-human DNA, mainly originating from the oral microbiome^19,20^. This contaminating DNA can negatively affect the proportion of human DNA in samples and the yield of usable human sequence. Recently, it has been demonstrated that bacterial reads found in saliva derived DNA in human whole genome sequencing can align to the human reference genome. This can result in the identification of false variants not present in the human genome^21^. We report that bacterial reads can also alter the genotype calls of true variants and present a solution to this problem through the use of a bacterial sequence alignment decoy.

## Results

### Identification of atypically aligned read pairs

**Figure 1:**
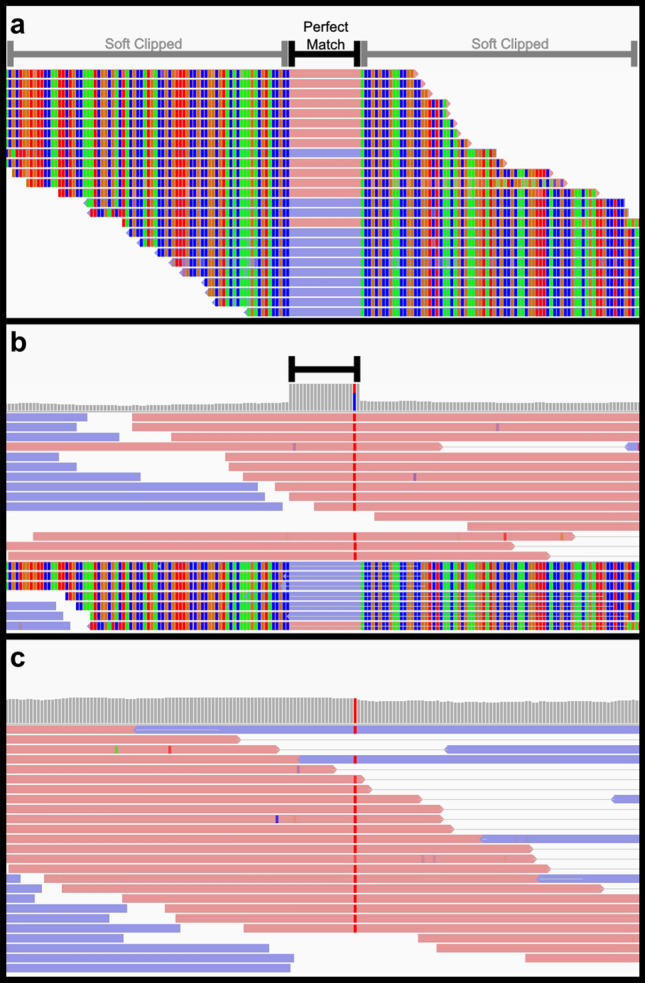
Atypical alignment of saliva-derived reads. Three example alignments are shown. (**a**) Atypically aligned reads with the core perfect match region indicated by the black bar and the flanking ‘soft clipped’ regions indicated with the grey bar. (**b**) The same incorrectly aligned reads are shown in context of a genome alignment with typically aligned reads displayed at the top of the alignment. The localised increase in depth can be seen in the histogram for the core perfect match region. A heterozygous C > T SNV identified by the variant identification software with high confidence (GQ = 99) can also be seen in this region where the reference allele is derived from the atypically aligned reads. (**c**) This C > T SNV is observed as homozygous alternate in a blood derived genome alignment from the same individual. Alignments are displayed in the integrated genomics viewer (IGV) in forward (pink) and reverse (purple), with alignment depth histograms in panes B and C. Mismatched and soft clipped bases are shown as green (A), red (T), orange (G), or blue (C) bands, with the same colour scheme used to indicate apparent SNVs in the depth histogram. Overlaps or horizontal lines between read alignments represent read pairs.

Atypically aligning read pairs (read pairs where the distance between the leftmost and rightmost mapped bases is at most 30 bp), were first identified following analysis of a subset of genomic variants detected in saliva-derived DNA samples. These variants were observed as clusters with heterozygous genotype calls and were unique to the proband (not observed in the ExAC database). These clusters were separated by 20 bp or less and contained mostly insertions of more than 10 bp (Supp. Table 1). Furthermore, the apparent variants had differential allelic depth ratios that strongly favoured the reference allele. However, the variants were not observed in alignments from whole exome sequencing for the probands’ parents. Analysis of alignments of the anomalous variant clusters using Integrative Genomics Viewer (IGV) revealed a subset of paired reads (20–50% at these sites) with unusual properties overlapping the variant locus (Fig. 1). These atypically aligning read pairs showed only a 19–25 bp region of sequence identity to the human reference genome (Fig. 1a). This region of identity was shared by both members of each read pair, resulting in overlapping alignments and a localised increase in read depth (Fig. 1b). The sequences flanking either side of the region of identity were soft clipped. These properties are consistent with the alignments found to cause false variant identification by Trost et al.^21^.

**Table 1:** Taxa distribution results from the BLAST algorithm for 8 saliva-derived genomes (internally and externally sourced).

Type	Internal A	Internal B	Internal C	ERX1462737 (22)	ERX1462740 (22)	SRX2830683 (23)	SRX2830684 (23)	SRX2830689 (23)
Archaea	6.42E−04%	3.84 E−04%	3.26 E−04%	8.65 E−03%	4.77 E−04%	3.22 E−04%	0.000%	2.03 E−04%
Bacteria	99.98%	99.95%	99.93%	58.80%	95.81%	99.91%	99.31%	99.74%
Fungi	1.03 E−03%	1.13 E−04%	1.43 E−03%	0.024%	0.020%	5.72 E−03%	0.022%	0.016%
Invertebrate	1.40 E−03%	7.01 E−04%	3.91 E−04%	0.032%	6.21 E−03%	4.27 E−03%	0.044%	4.81 E−03%
Plant	5.47 E−03%	2.58 E−03%	0.014%	40.710%	2.848%	3.83 E−03%	0.022%	0.153%
Protozoa	9.80 E−04%	1.65 E−03%	5.21 E−04%	0.013%	2.39 E−03%	3.79 E−03%	2.02 E−03%	1.15 E−03%
Human	5.41 E−04%	1.54 E−03%	6.58 E−03%	0.037%	0.032%	3.26 E−03%	0.079%	6.77 E−03%
Mammalian other	0.013%	0.012%	0.044%	0.197%	0.190%	0.061%	0.412%	0.066%
Vertebrate other	3.55 E−04%	0.019%	1.24 E−03%	0.121%	1.080%	5.72 E−03%	0.102%	9.55 E−03%
Viral	7.60 E−04%	0.015%	4.30 E−03%	0.058%	8.11 E−03%	1.65 E−03%	3.02 E−03%	1.08 E−03%

Interestingly, the anomalous variant clusters were predominantly identified immediately downstream of reference sequence regions identical with the atypically aligning read pairs. When insertions were identified, the apparent inserted sequence originated from the soft clipped region. In some regions de novo single nucleotide variants (SNVs) were called in the region of sequence identity to the reference genome. These SNVs generally had imbalanced allelic depth ratios, similar to the de novo variant clusters observed by Trost et al. Despite abnormalities in both the alignment and allelic depth ratio, variants within these anomalous read pair clusters were identified with average genotype quality (GQ) scores of 87.64 (from a set of 101 manually curated variants from internal genome A, range = 41–99, SD = 17.10).

Some SNVs identified within the region of sequence identity between the reference genome and atypically aligning reads appeared to be incorrectly genotyped. When we categorised reads by support for either the reference or variant allele, all atypically aligned reads supported the reference allele, while the typically aligned read pairs supported the variant allele (Fig. 1b). Therefore, in the absence of the atypically aligning reads, the locus would likely be genotyped as homozygous for the alternate allele, rather than the heterozygous genotype returned in the presence of the atypically aligned reads.

**Figure 2:**
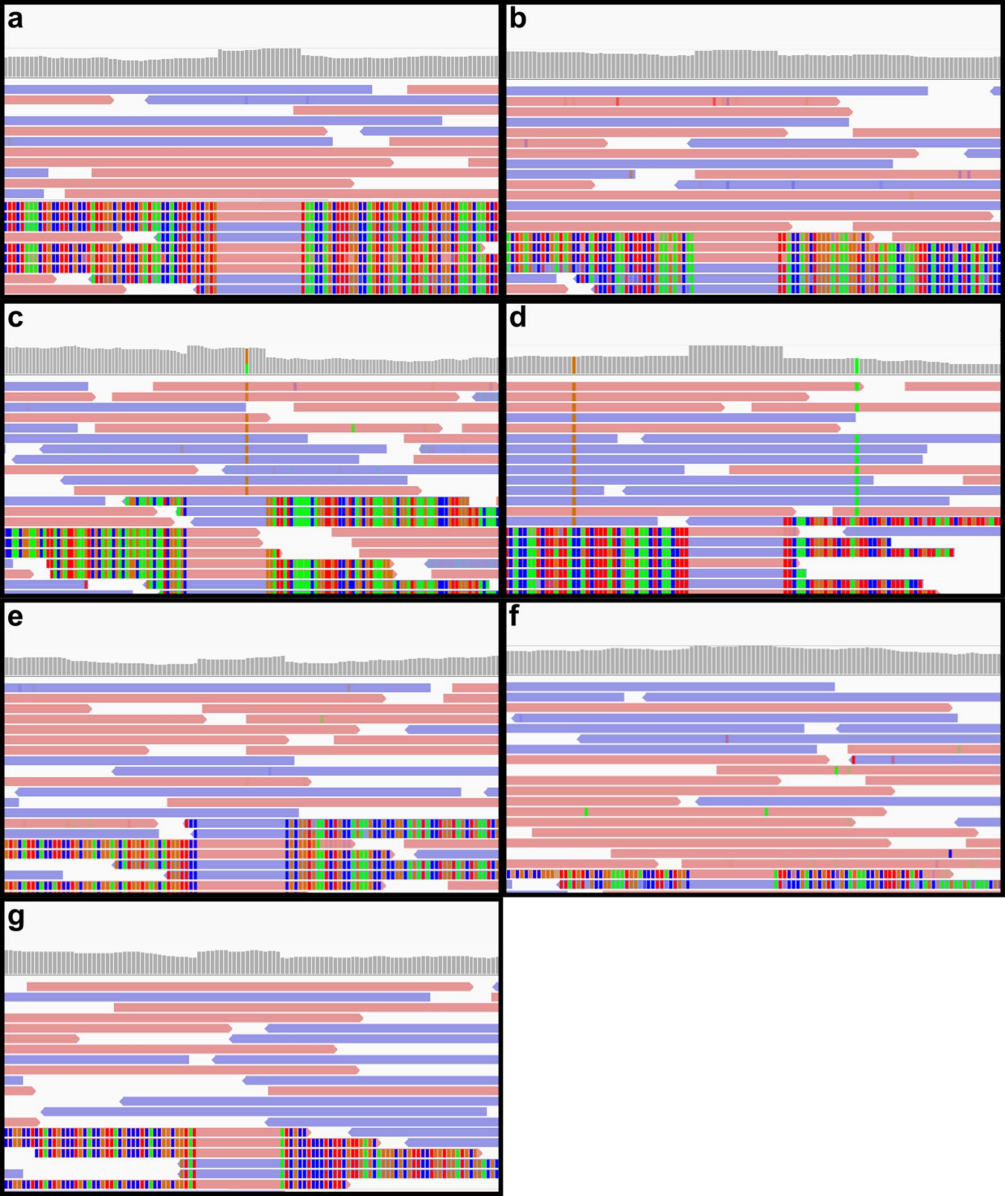
Examples of atypically aligned read pairs in multiple genomic alignments: (**a**) Internal B, (**b**) Internal C, (**c**) ERX1462737, (**d**) ERX1462737, (**e**) SRX2830683, (**f**) SRX2830684, and (**g**) SRX2830689.

Atypically aligned read pairs and associated identified variants were observed in all 18 individuals where whole genome sequencing (WGS) was performed using saliva-derived DNA. We also identified similar clusters in 5 publicly available saliva-derived human genomes (Fig. 2). Neither the atypically aligned read pairs nor the altered genotype calls were observed in 48 blood-derived in-house genomes. Therefore, we hypothesised the anomalous read pairs originated from the human oral microbiome.

### Atypically aligning reads are non-human in origin

We investigated the species origin of the atypically aligned reads from three in-house and five publicly available saliva-derived whole genomes^22,23^ by comparison to the GenBank RefSeq database^24^ using the BLAST algorithm. Atypically aligned reads were defined as read pairs where the distance between the leftmost and rightmost mapped bases of the pair (template length) was between 1 and 30 bp. This selected read pairs where both members of the pair mapped to a 30 bp region of the genome. For the majority of these reads (average of 63.46%), BLAST searches identified bacterial genera with highly similar sequences. The majority of highly similar sequences correlated with taxa found in the human oral microbiome (Table 1). There was still a significant proportion of the atypically aligned reads did not show significant similarity to any species in RefSeq (on average 32.86%).

Conversely, analysis of typically aligned reads by BLAST (from 1,000 randomly selected loci) resulted in 94.26% of reads matching *Homo sapiens* RefSeq sequences. The remaining proportion of reads without human matches showed similarity to other primate species (2.89%) or no significant similarity (2.1%) to RefSeq records.

Reads originating from food sources were present in two of the external saliva-derived genomes. Sample ERX1462740 showed a small portion of BLAST matches to plant (2.24% of matches) and non-mammalian vertebrate (1.08% of matches) genera while a large fraction of BLAST matches from sample ERX1462737 identified *Aegilops tauschii*, a progenitor of wheat (40.35%). This likely indicates recent consumption and potential non-compliance with collection instructions.

Of the atypically aligning reads that were compared to the RefSeq database, 45.41% had mapping quality scores of less than or equal to ten (range 25.08–52.40%), which are excluded by GATK HaplotypeCaller. However, 42.40% of atypical alignments had mapping quality scores of 40 or greater (range 30.65–63.17%) (Supp. Fig. 1). These atypical alignments are not excluded by GATK HaplotypeCaller and have the potential to confound variant identification. To confirm the effect of non-human reads on variant identification, we compared variants from saliva and blood derived sequences from the same individual. The atypically aligned reads and associated clustered variants were only observed in the saliva-derived sequence alignments and were absent in the blood-derived sequence alignments (Fig. 1).

In the WGS alignment from the saliva-derived sample, a proportion of variant loci with atypically aligned reads were called with heterozygous genotypes, while the same loci in the blood-derived sample were called as homozygous for the alternate allele. This alteration in genotype call was seen as the atypically aligned reads provided support for the reference allele resulting in a heterozygous genotype call.

In some instances, the affected loci overlapped with coding regions and the non-reference allele represented a nonsynonymous variant. The miscalling of variant genotypes as a result of atypically aligned reads has the potential for significant consequences, particularly when saliva-derived DNA is used for clinical diagnoses.

### A bacterial sequence decoy prevents atypical alignments

In order to sequester and suppress the alignment of non-human read pairs to the human reference sequence, we developed a human oral microbiome decoy sequence. The decoy comprised sequences from 77 bacterial genera, identified by BLAST searches of the unaligned reads from a single contaminated saliva-derived WGS sample (internal sample B) and setting a minimum threshold frequency of least 0.001% of these reads aligning (Supp. Table 2). BLAST results from un-aligned reads were used because the atypically aligned reads were too few in number to determine the full microbial diversity present in the sample, and may not represent all bacterial reads in the WGS pool. The complete genome sequences of the selected bacteria representing 77 identified genera (the most commonly observed species) were included in the decoy (or reference contigs when no genome was available).

To ensure that species contributing to atypically aligned reads were not excluded from the decoy, atypically aligning reads were compared to the RefSeq and NC databases using the BLAST algorithm. Seven species (representing at least 2% of positive BLAST results from this comparison) were added to the set of 77 selected bacterial genomes (Supp. Table 3).

After identification, sequences from 84 bacterial species were concatenated into a single decoy sequence separated by 1000 “N” bases. Regions of the decoy with sequence highly similar to the human genome (≥ 65% identity), polynucleotide repeats, and regions represented more than once in the decoy were masked by altering all bases in these regions to “N” bases. The resulting decoy was 203,185 kb long and is available for download from GenBank, accession MT169739.1.

**Figure 3:**
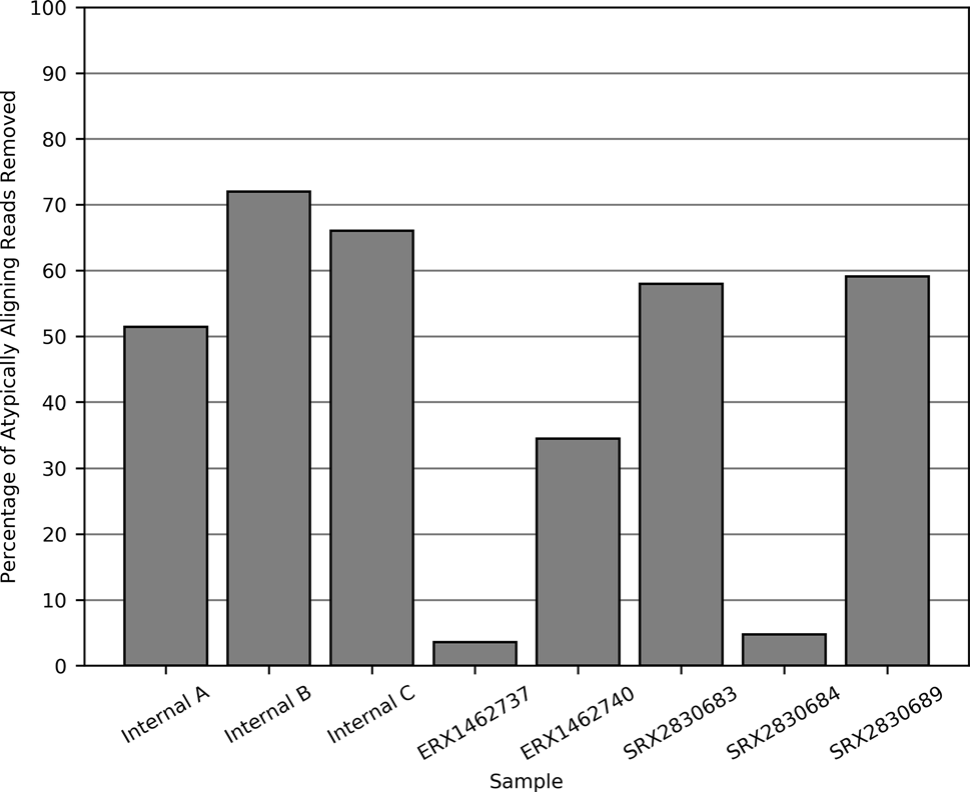
The proportion of atypically aligned reads (minimum mapping quality of 10) prevented from aligning to the human reference genome (GRCh37) by the incorporation of a bacterial decoy sequence in the genomic alignment. The use of the decoy had minimal effect in ERX1462737, a sample with significant plant contamination, and SRX2830684, a genome with very low levels of bacterial contamination.

**Figure 4:**
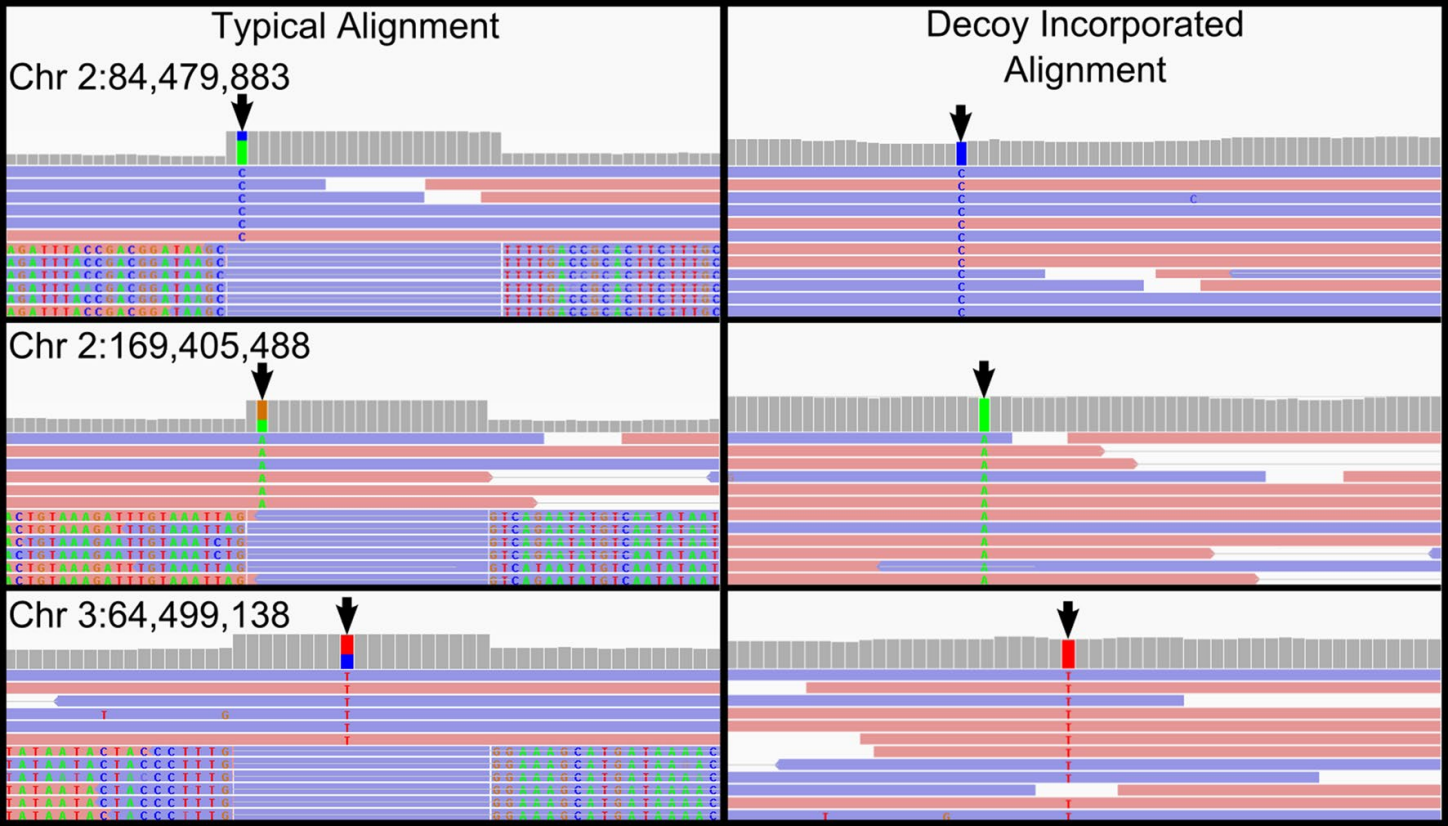
Effect of the bacterial decoy on variant identification by GATK HaplotypeCaller. Three loci are shown from internal sample A with genotype calls indicated by arrows before and after incorporation of the bacterial decoy. The left alignment shows SNVs called in heterozygous genotypes as a result of incorrectly aligned read pairs. The right alignment show those same sites after alignment to the decoy-supplemented genome.

The decoy was subsequently appended to the human reference genome for our in-house analysis (GRCh37). This extended reference was utilised for read mapping and variant discovery for all eight saliva-derived samples. Reads successfully mapped to the decoy and reduced the fraction of unaligned reads by an average of 44.84% (range 0.48–61.67% SD = 19.61). The use of the decoy also reduced atypical read alignments by an average of 43.66% (range 3.60–71.97% SD = 25.01) (Fig. 3), and resulted in identification of variants in correct homozygous alternate genotypes within these regions (Supp. Fig. 2). Importantly, with the use of the decoy, the genotypes from saliva-derived read alignments from internal sample A reflected the genotypes called in the blood-derived alignment (Fig. 4). Of note, the decoy performed poorly in two WGS saliva-derived datasets, ERX1462737 and SRX2830684, reducing atypically aligned reads by only 3.60% and 4.76%, respectively. The non-human reads in sample ERX1462737 originated predominantly from plants (outside of the scope of the bacterial decoy), while SRX2830684 contained very few atypically aligning reads, and likely had very little bacterial contamination. Excluding these two genomes, the average reduction of atypically aligned reads per saliva-derived genome was 56.83% (range 34.51–71.97% SD = 11.88).

As expected the decoy sequestered only a small fraction of reads mapped to human chromosomes in whole genome sequences derived from two in-house blood-derived DNA samples (internal D - 0.0069%, internal A - 0.0088%) and only a small proportion of variants were not identified when the decoy was added to the GrCH37 reference sequence (1.15% and 2.24 × 10^–3^% respectively). Variants that had not been called with the use of the decoy were of lower quality (median of 48.73 and 34.77 respectively) and the proportion of variants not identified was similar to that seen when comparing two variant identification replicates from the same genomic alignment (internal D - 1.27%, alternate calls were not available for the second sample).

DNA Genotek, a major manufacturer of saliva collection receptacles states that typically 10% of DNA extracted from saliva is bacterial in origin^25^. Many of the samples used in this research contained non-human DNA sequences substantially higher or lower than this. Three of the genomes analysed, ERX1462740, SRX2830689, and internal sample C had levels of contamination as predicted by the proportion of unmapped reads (4.14%, 7.11%, and 11.4% respectively). In these genomes 51, 894, and 1603 variants, respectively, were called at loci within 100 bp of potentially atypical alignments. These variants were not called when the decoy was incorporated. It was predicted based on the number of variants not called and the efficacy of the decoy in these three genomes that approximately 0.03% (an average of 0.00325%, 0.0330%, and 0.0550% respectively) of variants were called due to the presence of atypical alignments.

## Discussion

Trost et al. have recently reported that the presence of non-human reads in human genome sequencing data adversely affects variant identification^21^. They demonstrated that bacterial contamination contributes to an increased number of false-positive small variants identified, particularly rare single nucleotide variants. Our study extends these findings by demonstrating that bacterial reads can mislead state-of-the–art variant identification and genotyping algorithms to emit incorrect heterozygous genotypes for homozygous loci. Such spurious variants have the potential to adversely impact the accuracy of a diagnosis or confound research results where saliva was utilised as a DNA source.

Using the BLAST algorithm, we identified that the majority of atypically aligned reads (63.46%) were of bacterial origin. An additional 3.63% were derived from plant and animal species commonly consumed by humans (e.g. grains, vegetables, and fish), potentially reflecting food consumption prior to sample collection. In all eight saliva samples analysed, 32.9% of non-human reads showed no significant similarity to any sequences in the RefSeq and Nucleotide Collection datasets^24^ and may reflect microbial species which have yet to be described.

To prevent the alignment of non-human reads to the human reference sequence, we generated a decoy for sequences from oral bacteria by combining representative genomes for the most common bacterial genera observed in three in-house and five publicly available saliva samples. When including the combined sequence as an additional contig in our read alignment pipeline, we sequestered an average of 46.8% of non-human reads to the decoy and restored heterozygous genotype calls to their correct homozygous state.

The impact of contaminating microbial reads on human genome sequence alignment and variant identification is not due to algorithmic errors in the alignment or variant identification software. Aligners such as the Burrows-Wheeler aligner implicitly report all possible alignments that meet quality thresholds. Thus, all reads that can be aligned to the target reference sequence are reported. Additionally, GATK HaplotypeCaller and other variant identification software expect that the alignment is derived exclusively from reads of the same species as the reference sequence. It is possible that fine-tuning of alignment and calling parameters could prevent contaminating reads from aligning to the human reference and incorrect identification of variants. However, this would require optimisation for each individual sequenced and therefore, could not be easily used for large numbers of samples. It would also complicate comparisons of variants identified from different samples and/or projects.

In samples with ‘typical’ levels of bacterial contamination (4.14–11.4%) we estimated that approximately 0.03% of variants were called due to the presence of atypical alignments. We expect that a similar proportion will be observed in similarly contaminated saliva-derived sequencing sets. Events were observed where atypical alignments overlapped with homozygous SNVs. In these cases the variant was correctly identified, but with an incorrect heterozygous genotype. Such events were infrequent and could only reliably be detected by manual analysis preventing an accurate measure of their prevalence. We predicted that a similar rate of variants were called due to the presence of atypical alignments when analysis was limited to coding variants (an average of 0.012%, ERX1462740 was excluded as only two variants were removed). However, a larger dataset is required to determine if atypical alignments have the same effect in coding and non-coding loci. As the alignment of non-human reads appears to be driven by random regions of identity between reads and the reference, we expect that all loci and genes have the potential to be affected by atypical alignments.

The bacterial decoy developed in this study sequestered over 40% of the atypically aligned read pairs in eight saliva-derived genomes analysed, without observed impact on variant identification sensitivity and specificity. Expanding the scope of the decoy by expanding the diversity of bacterial genera was attempted, but exponentially diminishing returns in performance were observed (data not shown). Plant and animal genomes are generally too large to be effectively used as a decoy (due to computational memory requirements during alignment), and animal-derived decoys also increase the risk of sequestering human-derived reads originating from conserved genome regions. Therefore it is unlikely that an expanded decoy to capture potential food-based contamination would effectively capture additional contaminating DNA sequences without negatively affecting alignment rate and computational efficiency. However, improvements in the optimisation of the decoy to only include the sequences from the regions of bacterial genomes similar to the human genome, would improve the utility of the decoy. Additionally, a wide species diversity is seen in human oral microbiota composition due to multiple factors including diet and antibiotic use^26,27^. This diversity may necessitate the development of individual decoys tailored to commonly observed oral microbiota compositions based on particular populations or food preferences.

None of the atypical read pairs observed in this study aligned to more than 24 bp of the human reference genome, therefore, alteration of the seed length of BWA from the default of 19 bp to a higher value, such as 25 bp would prevent the alignment of these reads. However, such a change in alignment parameters would affect comparisons to previously published variant data and reduce the alignment quality in highly variable regions of the genome.

A further option is to remove or annotate read alignment records based on the total aligned length of read-pairs by direct manipulation of the SAM ‘TLEN’ field. This method could remove reads without computationally expensive re-alignments. However, it would likely remove a small fraction of reads of human origin, which may have an adverse effect on variant identification. The most straightforward implementation of this would be to filter aligned read pairs on the template length field of the SAM/BAM file format, although this relies on both members of a read pair aligning to the reference genome. Filtering reads based on alignment length is the only method that can be performed after the alignment process. However, alignment filtering would require an additional processing step, and repeating computationally expensive variant discovery and genotyping. Additionally, filtering alignments will affect comparability to established variant databases.

While the bacterial component of DNA extracted from saliva causes atypical alignments in whole genome sequencing data, we do not expect the same phenomenon to occur in the analysis of exome sequence data. Whole exome sequencing includes the selective hybridisation of sample DNA to oligonucleotide probes to enrich exonic DNA regions. It is unlikely bacterial DNA will efficiently bind to the oligonucleotide probes and thus will not be present in the sequencing library. In addition, the atypical alignments appear to be due to random identity to the reference genome rather than any particular genomic features. Therefore, the phenomenon we describe will likely be observed when the data is mapped to other builds of the human reference genome. This is supported by our observation that 99.95% of atypical alignments in GRCh37 regions were also included in the GRCh38 assembly.

Despite the multiple methods to prevent non-human reads impacting variant identification, there is currently no obvious solution that is scalable whilst retaining comparability to previously generated data. Due to the lack of a clear solution we suggest that a bacterial decoy, such as the one presented here, is used when processing whole genome sequencing data generated from saliva-derived DNA. Additionally, as use of saliva-derived DNA for genomic sequencing becomes more popular, it must be ensured that variants affected by non-human contamination are not integrated into population databases.

## Conclusion

Short sequence reads of non-human origin can align to the human reference sequence under current best-practice alignment parameters, resulting in false-positive variant and incorrect genotype calls. Decoy sequences can successfully sequester non-human reads, preventing them from aligning to human sequences. However, no obvious solution exists for the complete removal of all observed non-human reads. Therefore, where possible, blood should be used for the DNA source for whole genome sequence variant discovery until alternate methods for the removal of non-human DNA or reads become available. Where blood cannot be used as a DNA source a decoy, such as the one presented here, should be used to minimise the impact of non-human reads on alignment and variant identification.

## Methods

### Genome re-sequencing

Publicly available genomes were sourced from the NCBI short read archive^24^. Search parameters were limited to publicly available human genomic DNA sequencing records returning the search term ‘saliva’ or ‘oral’. Whole genome sequencing data for two cases from the Simons Genome Diversity Project were selected for use in saliva decoy testing (ERX1462737 and ERX1462740). Both datasets consisted of 101 bp paired end reads from libraries prepared using the Illumina TruSeq PCR free preparation kit^22^. Additionally, publicly available 100 bp paired end reads, from a parents/child trio were selected for use in saliva decoy testing. The libraries were prepared using PCR amplification (SRX2830683, SRX2830684, and SRX2830689)^23^. All five of samples were sequenced using the Illumina HiSeq 2000 platform at an average depth of 40 × and the raw reads were downloaded as paired-end fastq files from the NCBI short read archive. This data was selected because it was comparable to our own whole genome data set (151 paired end HiSeq X Ten sequencing at 30 × depth of libraries prepared using the Illumina TruSeq PCR free preparation kit)^28^.

### Genome alignment and variant identification

Reads were processed in accordance with GATK3 best practice. Alignment to the GRCh37 reference genome was performed using the BWA-mem algorithm (version 0.7.17)^29^, followed by de-duplication by Picard MarkDuplicates (version 2.1.0). Variant identification and joint genotyping were performed using the GATK (version 3.7) ‘Haplotype Caller’ and ‘GenotypeGVCFs’ algorithms^30^, as described previously^31^.

### Blast

Query sets were generated by selecting aligned reads with an observed absolute template length between 1 and 30. BLAST searches were performed against the NCBI RefSeq database as of January 1st 2019 using the entire read sequence. Results were filtered to only include those with at least an 80% coverage of the query read and an e-value maximum of 1. Results were tallied based on species and genera.

### Ethics approval and consent to participate

Whole genome sequencing of in-house samples was approved by the New Zealand Northern B Health and Disability Ethics Committee (ref 12/NTB/59), and parents provided written informed consent. All methods were performed in accordance with the guidelines and regulations outlined by this approval.

### Consent for publication

Not applicable.

## Supplementary information

Supplementary Information

## Data Availability

The in-house WGS datasets are not consented for public data deposition, but identified variants are available from the corresponding author on request. Externally acquired WGS data was obtained from The Short Read Archive (SRA) under the following entries: ERX1462737, ERX1462740, SRX2830683, SRX2830684, and SRX2830689. The decoy sequence can be downloaded from the GenBank repository under accession MT169739. The BLAST algorithm results can be found in the supplementary materials.
