# Supplementary material for: Contaminating DNA in human saliva alters the detection of variants from whole genome sequencing
Source: Sci Rep. 2020 Nov 6;10:19255. doi: 10.1038/s41598-020-76022-4 (PMC7648094; doi:10.1038/s41598-020-76022-4)
Supplement: Supplementary file 1 — Supplementary Information [file 41598_2020_76022_MOESM1_ESM.pdf]

Contaminating DNA in Human Saliva Alters the Detection of Variants from Whole Genome Sequencing.

Samson CA, School of Biological Sciences, The University of Auckland, Auckland, New Zealand. csam488@aucklanduni.ac.nz

Whitford W, School of Biological Sciences, The University of Auckland, Auckland, New Zealand.

Snell RG, School of Biological Sciences, The University of Auckland, Auckland, New Zealand.

Jacobsen JC, School of Biological Sciences and Centre for Brain Research, The University of Auckland, Auckland, New Zealand. j.jacobsen@auckland.ac.nz *

Lehnert K, School of Biological Sciences, The University of Auckland, Auckland, New Zealand.

*Corresponding author

Supplementary Table 1- Examples of anomalous variant calls which lead to the identification of atypically aligning reads. Variants displayed are derived from the alignment of internal sample A

Chromosome	Position	Reference allele	Alternate Allele	Genotype	Allelic Depth Ratio (Ref,Alt)	GQ Score
**1**	1443328	C	CCGCGTGCGCCGTGAGCGCATGGGTCACATCGACCTGGCCG	0/1	30,3	78
**1**	3019209	T	TCGTTTCGCCATGCACTCTGCCTATTTGCGCCATCTTATCAAAGGCGAGAGCGTCTTCTTGGTGAAAGACGGCGTTATCAAATTTAAGAACTTCAAGCGTA	0/1	24,3	53
**1**	3431499	G	GTACGCGCCTTCCTTAGCGAAAACCGCACGGACAGTACGCAGAAGAATAATGAAGTCACCGGTAAGCGACCAGTTCTCAACATAGTAGAGATCCAGACGAACCGACTCTTCCCAAGAGAGATCA	0/1	25,3	49
**1**	4213486	T	TCCGCACTGGCTCAATTTTTGGGCACAACGTTTGAATATCGCGATTAAAAAATCAGTGTTTGAACCGGATATAAAAAGGCCGTCTGAAATCAAGGTTTCAGACGGCCTGCTATTTTCAAATCAAGAAGGA	0/1	29,11	99

**1**	4282539	G	GCGCTACTGAGGGCAGCGTTTTTGCCGCCGTCACGGATAATGCCGAG CACGTAGGCTCGCA	0/1	31,6	99
**1**	4282543	C	CGTTGA	0/1	31,6	99
**1**	4282545	C	CTCAATCATGTTCCGCCCGGGCTCAGTACTGACCCCTAGCTCTTTCGC G	0/1	29,6	99
**1**	5509136	A	ATCGCAGCCGCACCGGCTACGATTTCATTT	0/1	24,3	53
**1**	5509138	A	ATTCATTTGTGATACTTGCTTGACGAGCTTTGTTATACACCAACCGCA GATCATTAATTAAATTACCTGCAT	0/1	23,3	53
**1**	6392150	C	CATCGCCTCCCTCTTCGTTGAG	0/1	18,3	67
**1**	6392152	A	AGG	0/1	19,3	69
**1**	6392155	G	GCATTCCTGGTGGATGCGGACGCTATCTCCCGCTCCCTCATGGAGCCC GGCGAGGCGGTGCTCGCCCGCACGGTCGCGGAGTTCGGTGAGCA	0/1	19,3	69
**1**	7830320	T	TCGCGCTGGCTG	0/1	28,12	99
**1**	7830322	C	CCGTTCGTTGACCTTGCCCGGCGCAATGGAGGG	0/1	29,12	99
**1**	7830325	T	TGGCATTTTTGTTGAAACCGGATTGGTCGTATTTGAAACCGGGTACG ATGCTGACTG	0/1	31,12	99
**1**	8579657	A	C	0/1	24,3	54
**1**	8579660	C	CAGCCTGGTTCAACAACACGAAGTCCGCTGGACTTGGGTAAAAGGCC ATGCCGGACACCCTGAAAACGAAAAAGCCGACGAGCTCGCCAACCG AGGCGCAGCAAAATTTTT	0/1	24,3	54
**1**	8658064	G	GTTTTTA	0/1	36,11	99
**1**	8658066	C	CGTGTTATTGCCCTA	0/1	37,11	99
**1**	8658069	T	TGAAAATCTTTTTACCATATTGGATATAATCCAA	0/1	37,10	99
**1**	8658073	C	A	0/1	39,9	99
**1**	8658076	C	CTTAA	0/1	38,9	99
**1**	8658078	C	CGCACTGCAAAATTACGAAATATATATGGCAACAACCAACAATTTCAA AATATT	0/1	38,9	99

Supplementary Table 2- Top 77 genera used for decoy development

Species	Number of Sequences Returning with Match	% of Submitted Sequences	Accession of Sequence(s) Used
Prevotella	56417122	22.78228	NZ_CP013195.1
Neisseria	24623277	9.943334	NC_003112.2
Streptococcus	17799602	7.187808	NC_015678.1
Haemophilus	11684583	4.718451	NC_000907.1
Veillonella	8964212	3.619915	ACIK02000001.2-ACIK02000025.2
Rothia	5967605	2.409829	NC_013715.1
Actinomyces	5504468	2.222806	NZ_CP012590.1
Porphyromonas	5503687	2.222491	NC_010729.1
Eubacterium	3554132	1.435224	NZ_CP012068.1
Fusobacterium	3470611	1.401497	NC_003454.1
Campylobacter	3267852	1.319619	NZ_CP012541.1
Granulicatella	2538844	1.025232	NZ_KI391971
Atopobium	2138896	0.863726	NC_013203.1
Gemella	2052068	0.828663	NZ_CP014233.1
Leptotrichia	1945440	0.785605	CP014231.1
Oribacterium	1515533	0.612	ACKX01000001.1-ACKX01000254.1
Capnocytophaga	1515252	0.611887	NZ_CP012589.1
Solobacterium	1506910	0.608518	AECQ01000001.1-AECQ01000066.1
Selenomonas	1380182	0.557343	NZ_CP012071.1
Lautropia	1010801	0.40818	AEQP01000001.1-AEQP01000030.1
Megasphaera	875359	0.353486	AECS01000001.1-AECS01000049.1
Peptoniphilus	812443	0.32808	PNGX01000001.1-PNGX01000004.1
Achromobacter	481071	0.194265	NZ_LN831029.1
Aggregatibacter	462896	0.186926	NZ_CP012067.1
Corynebacterium	454905	0.183699	NZ_LN831026.1
Candidatus	363746	0.146887	NZ_CP007496.1
Lachnospiraceae	264750	0.106911	NBBL01000001.1-NBBL01000017.1
Catonella	236983	0.095698	ACIL03000001.3-ACIL03000026.3
Eikenella	152940	0.06176	NHRS01000001.1-NHRS01000006.1
Treponema	139123	0.05618	NZ_CP009228.1
Cardiobacterium	139052	0.056152	FKLO01000001.1-FKLO01000088.1
Kingella	125553	0.050701	AEWV01000001.1-AEWV01000057.1
Tannerella	108155	0.043675	CP017038.1
Lactobacillus	55357	0.022354	NC_013198.1
Abiotrophia	51159	0.020659	ACIN03000001.3-ACIN03000020.3
Peptostreptococcus	46552	0.018799	FOVA01000001.1-FOVA01000060.1
Dialister	45854	0.018517	GG698602.1

Lachnoanaerobaculum	35132	0.014187	AEPW01000001.1-AEPW01000150.1
Propionibacterium	33231	0.013419	NC_006085.1
Parvimonas	19673	0.007944	NZ_CP009761.1
Bacteroides	17869	0.007216	BAIU01000001.1-BAIU01000095.1
Mogibacterium	17277	0.006977	JALU01000001.1-JALU01000030.1
Centipeda	16449	0.006642	AFHQ01000001.1-AFHQ01000072.1
Pseudomonas	16277	0.006573	NC_002516.2
Bacteroidetes	15771	0.006369	NVWV01000001.1-NVWV01000022.1
Mycoplasma	15486	0.006254	NC_000912.1
Simonsiella	14083	0.005687	ADCY02000001.2-ADCY02000082.2
Klebsiella	12982	0.005242	NC_016845.1
Enterococcus	11871	0.004794	NC_004668.1
Actinobaculum	10155	0.004101	AWSB01000001.1-AWSB01000117.1
Staphylococcus	8968	0.003621	NC_004461.1
Finegoldia	8754	0.003535	NC_010376.1
Mitsuokella	7349	0.002968	ABWK02000001.2-ABWK02000031.2
Johnsonella	7300	0.002948	ACZL01000001.1-ACZL01000080.1
Eggerthia	7203	0.002909	ACZL01000001.1-ACZL01000080.1
Bifidobacterium	7003	0.002828	NZ_AP012324.1
Bulleidia	6923	0.002796	ADFR01000001.1-ADFR01000016.1
Olsenella	6791	0.002742	CP012069.2
Peptostreptococcaceae	6427	0.002595	AWVB01000001.1-AWVB01000160.1
Filifactor	6208	0.002507	NC_016630.1
Escherichia	5763	0.002327	NC_002695.1
Bacillus	4668	0.001885	NC_003997.3
Shuttleworthia	4517	0.001824	ACIP02000001.2-ACIP02000010.2
Gardnerella	4261	0.001721	NC_014644.1
Lactococcus	4257	0.001719	NC_002662.1
Eggerthella	4138	0.001671	AGEJ01000001.1
Stenotrophomonas	3691	0.00149	NC_010943.1
Listeria	3284	0.001326	NC_003210.1
Scardovia	3261	0.001317	AKCI00000000.1
Anaerococcus	3033	0.001225	NC_013171.1
Lysinibacillus	2993	0.001209	NZ_CP010820.1
Anaeroglobus	2816	0.001137	AGCJ01000001.1-AGCJ01000103.1
Gracilibacteria	2812	0.001136	PDRB01000001.1-PDRB01000010.1
Moraxella	2745	0.001108	NZ_CP014234.1
Enterobacter	2597	0.001049	NZ_CP025225.1
Desulfobulbus	2594	0.001048	NZ_CP021255.1
Bordetella	2586	0.001044	NC_002929.2

Supplementary Table 3- Species representing at least 2% of BLAST results for atypically aligning reads

Species	Number of Sequences Returning with Match	% of Submitted Sequences	Accession of Sequence(s) Used
Prevotella jejuni	69100	25.22634	NZ_CP023863.1-NZ_CP023864.1
Prevotella intermedia	35311	12.89099	NZ_CP019300.1-NZ_CP019301.1
Haemophilus influenzae	15001	5.476416	NC_000907.1
Capnocytophaga sputigena	9521	3.475832	NZ_CP022385.1
Prevotella scopos	8342	3.045415	NZ_CP016204.1-NZ_CP016206.1
Rothia aeria	6759	2.467509	AP017895.1
Neisseria elongata	5914	2.159025	NZ_CP007726

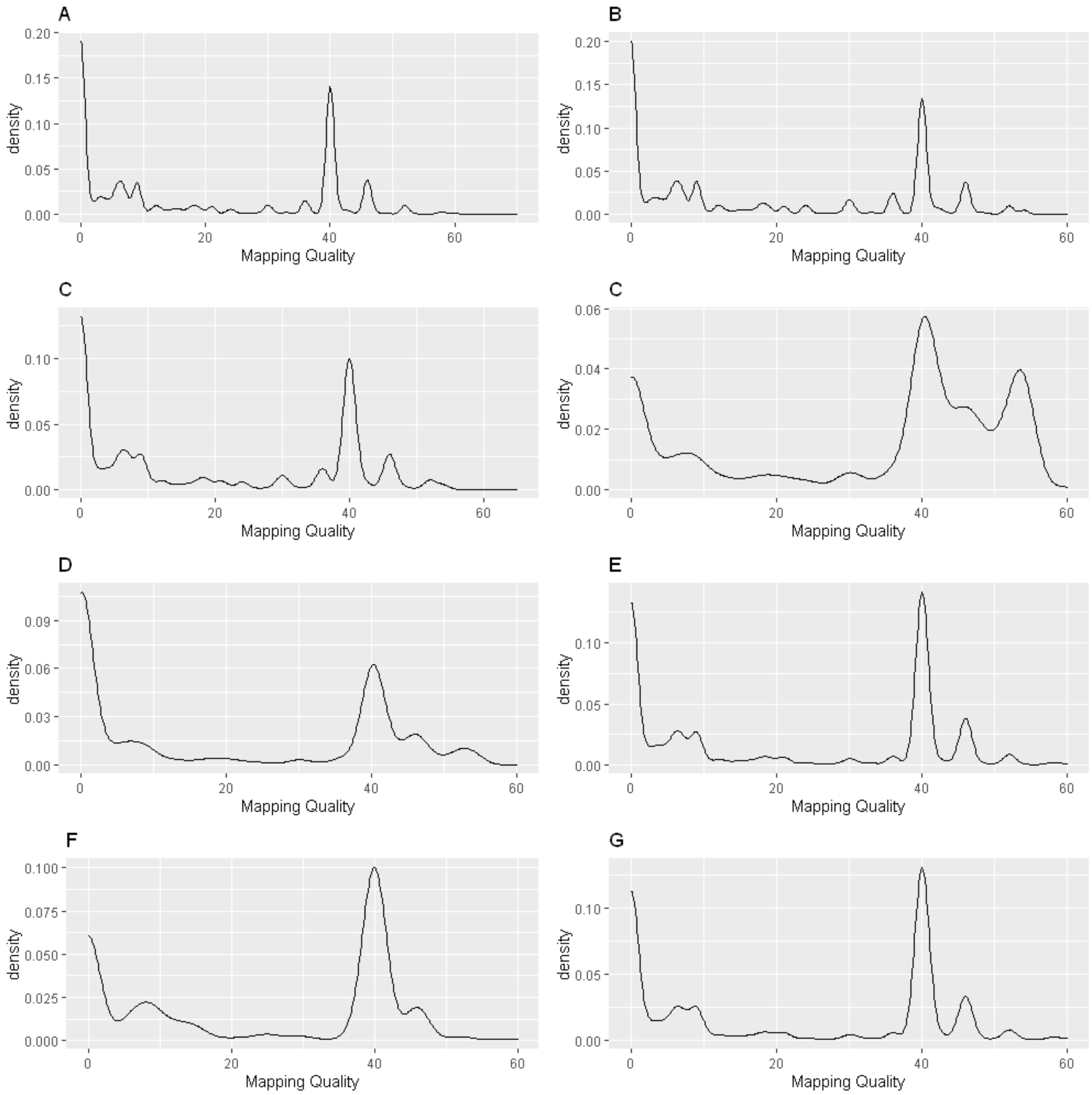

Supplementary Figure 1 – The distribution of mapping qualities of atypically aligning reads. For all samples density peaks are seen in the distributions as 0 and 40. The distributions of internal sample A (A), internal sample B (B), internal sample C (C) ERX1462737 (D), ERX1462740 (E), SRX2830689 (F), SRX2830684 (G), and SRX2830689 (H) are shown.

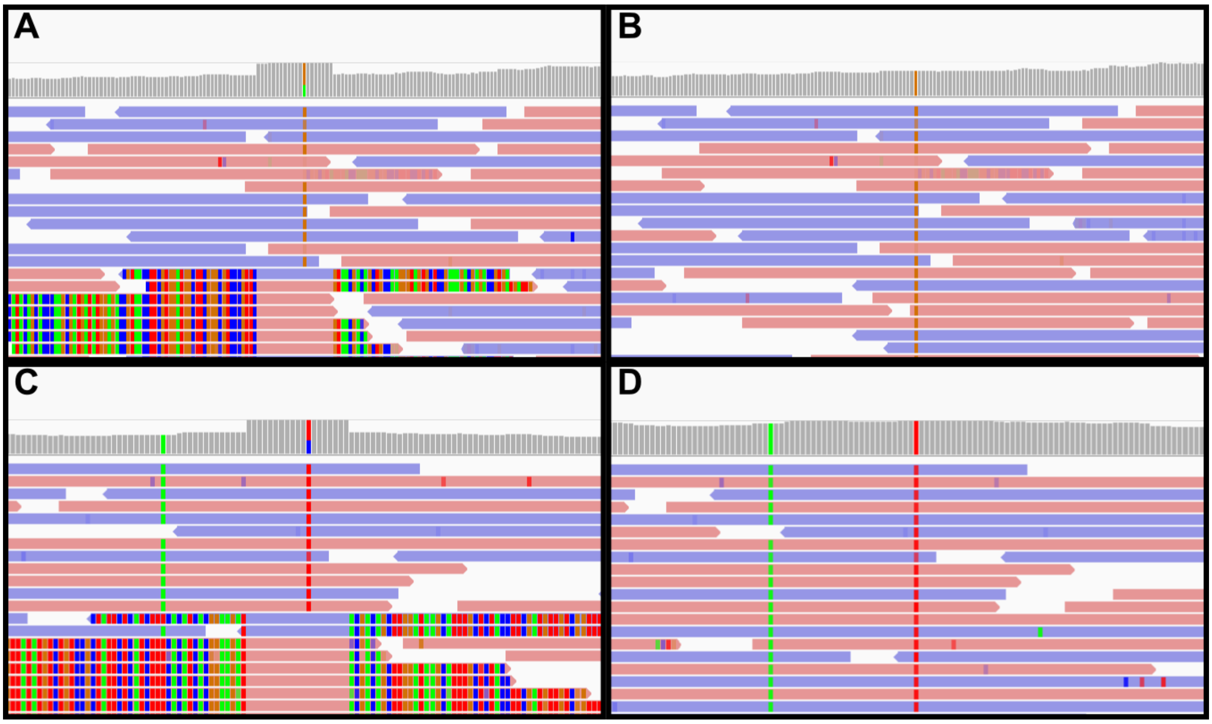

Supplementary Figure 2 – Examples of SNVs altered by the presence of atypical read pairs. A heterozygous, non-coding, SNV (A>G) in internal sample B (A) is called as homozygous when the atypical pairs are removed (B). The same effect is observed in SRX2830689 for a coding SNV (C-D).
